# Rapid expansion of the ascending aorta following fluoroquinolone therapy

**DOI:** 10.1016/j.xjtc.2025.07.004

**Published:** 2025-07-18

**Authors:** Takuro Makiura, Masahiro Daimon, Sachiko Kanki, Takahiro Katsumata

**Affiliations:** Department of Thoracic and Cardiovascular Surgery, Osaka Medical and Pharmaceutical University, Takatsuki, Osaka, Japan

**Figure:**
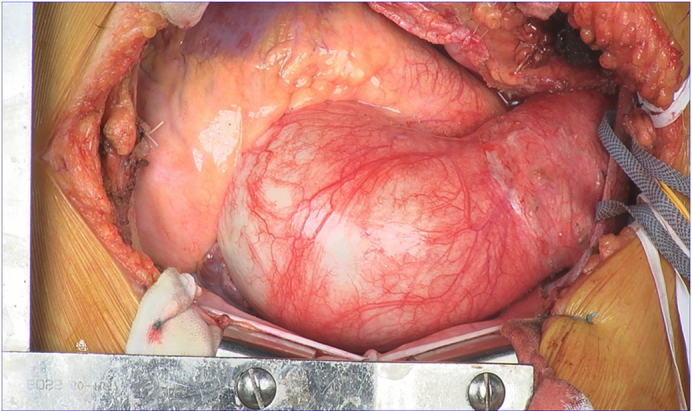
This is an AA following fluoroquinolone therapy over the span of 3 years.

**Central Message**

Fluoroquinolones can contribute to the development of aortic aneurysms and dissections and should be used carefully. If they are prescribed, patients must be monitored for rapid aortic enlargement.

Fluoroquinolones are antibiotics that are widely used in clinical settings. However, they can influence connective tissue and degrade the extracellular matrix, potentially weakening aortic walls. In addition, certain high-risk groups are more susceptible to the adverse effects of fluoroquinolones. Individuals older than age 35 years; women; and patients with hypertension, atherosclerotic lesions, a history of smoking, a family history of aortic disease, or a medical history of aortic aneurysms (AAs) or aortic dissections (ADs) are reportedly at increased risk of developing an AA or AD. Herein, we report a case where fluoroquinolone administration may have contributed to the development of a thoracic AA.

## Case Report

**Figure E1:**
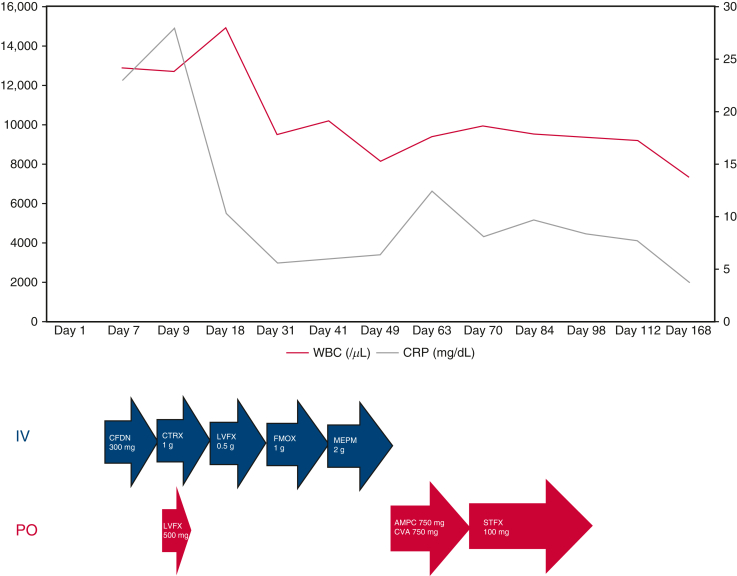
This chart illustrates the relationship between the patient's white blood cell count/C-reactive protein (*WBC*/*CRP*) trends and antibiotic treatment. WBC and CRP levels showed significant improvement with administration of fluoroquinolone antibiotics. However, after the antibiotics were switched out, WBC and CRP levels increased again, so fluoroquinolone therapy was reinitiated. Because WBC/CRP levels showed a downward trend and symptoms were alleviated, the patient's care was managed using follow-up observation. *CFDN*, Cefdinir; *CTRX*, ceftriaxone; *LVFX*, levofloxacin; *FMOX*, flomoxef; *MEPM*, meropenem; *AMPC*, amoxicillin; *CVA*, clavulanate; *STFX*, sitafloxacin.

A 72-year-old woman with a history of hypertension and malignant lymphoma, in remission for 27 years, visited our hospital. She did not have any history of smoking or family history of cardiovascular disease. Three years ago, she presented with fever and joint pain and received antibiotic treatment, including fluoroquinolones, at another hospital. Because of her persistent symptoms, she was referred to our institution for further evaluation and management. Even with the continuation of the antibiotic therapy, our extensive investigations failed to identify a definitive cause for her fever. Her symptoms were resolved after a total of 53 days of fluoroquinolone administration (Figure E1).

**Figure 1:**
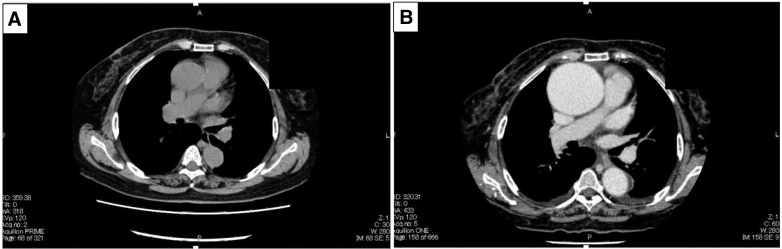
Three years ago (A) and just before surgery (B), preoperative computed tomography image showed that the diameter of the aorta was 41 mm and 71 mm.

Approximately 3 years later, she returned to our hospital complaining about palpitations and shortness of breath. A chest and abdominal computed tomography (CT) scan revealed rapid enlargement of the ascending aorta and thoracoabdominal aorta where the diameter had increased from 41 to 71 mm and from 25 to 35 mm, respectively, over a 3-year period, as well as moderate aortic regurgitation (Figure 1). Laboratory findings were unremarkable, and there were no clinical signs of infection. A carotid ultrasound revealed severe carotid artery tortuosity. Meanwhile, chest and abdominal CT scans did not show any evidence of aortic/systemic infections or anatomical abnormalities except for the AA. Minimal aortic wall calcification was also observed.

**Figure 2:**
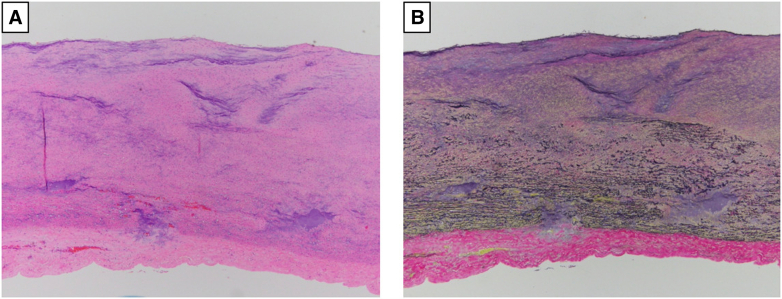
Hematoxylin-eosin staining (A) and elastica van Gieson staining (B) revealed the loss of medial elastic fibers extending from the aneurysmal region to the distal aortic arch.

**Figure E2:**
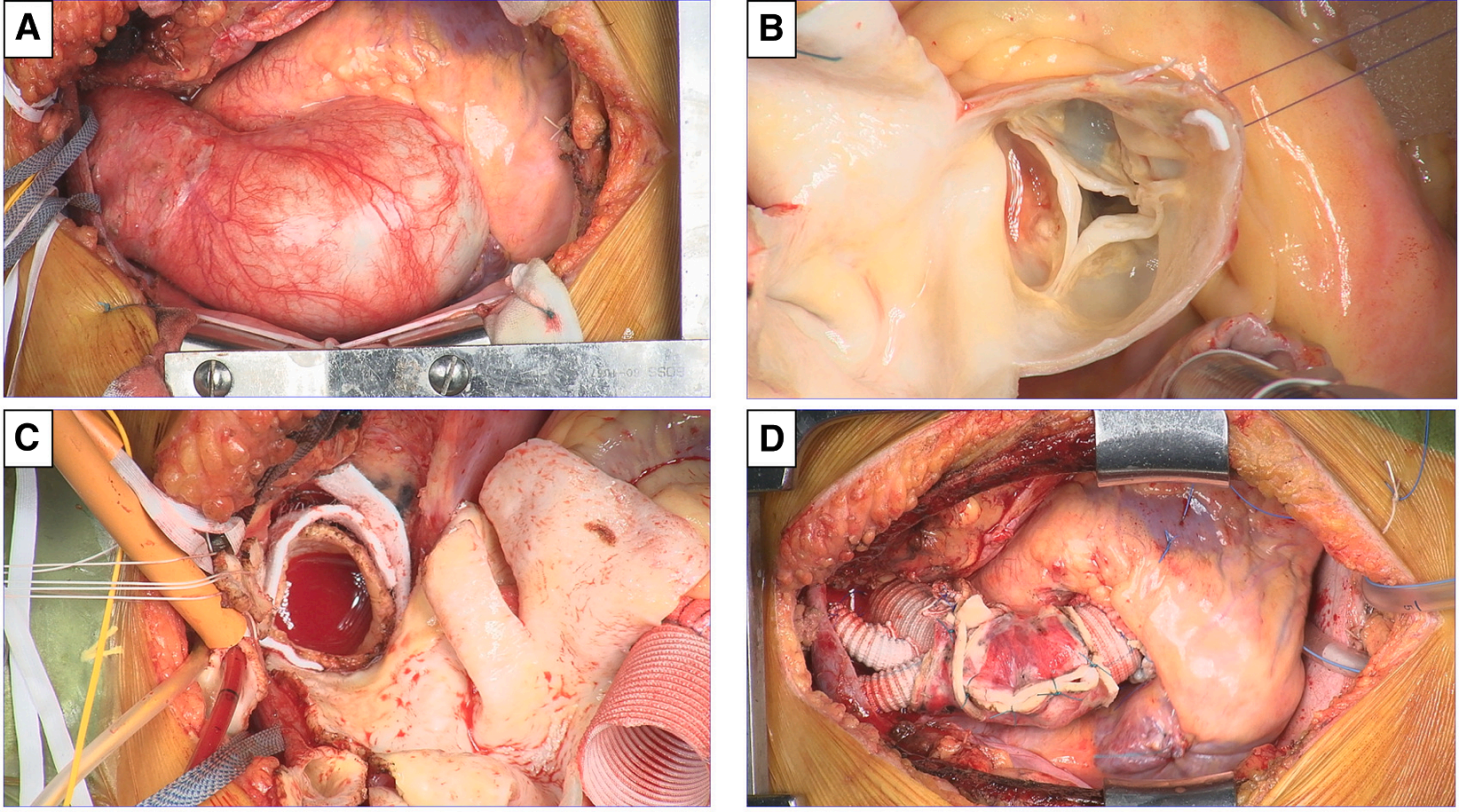
Intraoperative findings. A, A median sternotomy revealed an ascending aortic aneurysm. B, After cardioplegic arrest was induced during a cardiopulmonary bypass procedure, the aortic valve was examined. The tricuspid valve showed mild cusp prolapse and degeneration. C, After the aortic valve was replaced using a prosthetic graft, the aorta was incised during hypothermic circulatory arrest. The distal aneurysmal segment was then resected and reconstructed. D, The prosthetic graft was anastomosed to the distal aortic arch, after which the cardiopulmonary bypass procedure was continued. The graft was sequentially anastomosed to the proximal aorta and cervical branch arteries using selective antegrade cerebral perfusion.

Due to the presence of symptomatic aortic regurgitation and rapid aortic expansion, the patient underwent ascending and aortic arch replacement with a branched graft, as well as aortic valve replacement. There were no inflammatory changes, signs of infection, or adhesions in the outer layer of the aorta. The inner layer of the aorta was smooth with no visible entry points or scars (Figure E2). Her surgical recovery was uneventful, and she was discharged on postoperative day 23 after she had achieved independent ambulation. Pathological examination of the resected aorta revealed the loss of medial elastic fibers extending from the aneurysmal region to the distal aortic arch, but no elastic fiber rupture or false lumen was observed (Figure 2). Genetic testing for hereditary connective tissue disorders was performed using peripheral blood leukocytes during outpatient follow-up, but no pathogenic mutations were identified. To date, 1 year after surgery, the patient has not experienced any recurrence of the AA, and her recovery continues to be favorable.

## Discussion

Fluoroquinolone exposure can reportedly induce mitochondrial dysfunction in human aortic smooth muscle cells, promote apoptosis, and activate the mitogen-activated protein kinase pathway, thereby destabilizing elastic fibers and collagen. This can potentially trigger the development of AAs and ADs in the presence of inflammatory stimuli.^1^ In brief, individuals older than age 35 years; those who are women; and those with atherosclerotic lesions, a history of smoking or hypertension, a family history of major vascular disease, or a medical history of aortic enlargement/AD^2^ are at increased risk for fluoroquinolone-associated aortic disease. Animal experiments in mice regarding aortic enlargement caused by fluoroquinolones have reported that the risk of AA and AD is highest in the ascending aorta, followed by the aortic arch, thoracoabdominal aorta, descending thoracic aorta, and infrarenal abdominal artery. This may also apply to human beings, who are members of the same mammalian species.^E1^

A previous case-crossover study indicated that the risk of aortic events increases with longer fluoroquinolone exposure.^3^ However, an estimated 7246 patients would need to be treated with fluoroquinolones to induce 1 case of AA/AD, suggesting that these events are relatively rare.^E2^ Some reports have also suggested that the infections for which fluoroquinolones are initially prescribed can independently increase the risk of AAs/ADs.^E3^ In contrast, other studies have not demonstrated any clear association between AAs/ADs and fluoroquinolone use when it is limited to the treatment of single-organ infections or prophylaxis.^E4^ Additionally, fluoroquinolones can be more beneficial than other antibiotics because they have high tissue bioavailability and are broad-spectrum antimicrobial agents, which makes them particularly effective against refractory or life-threatening infections. Therefore, when fluoroquinolones are clinically indicated for treatment, their administration should not be withheld due to concerns about adverse aortic events, as our current case demonstrates.

Our patient had multiple risk factors for fluoroquinolone-associated AAs, including being a woman and older, with hypertension and mild aortic dilation. Three years ago, she had received prolonged fluoroquinolone therapy, spanning approximately 1.5 months. When antibiotic treatment was initiated, CT scans showed only mild aortic dilation, whereas a follow-up CT scan revealed rapid enlargement of the ascending aorta 3 years later. The patient's family history and physical findings did not indicate the presence of an AA/AD or hereditary connective tissue disorders, and she also had no history of smoking. Furthermore, genetic panel testing did not identify any pathogenic mutations. However, pathological examination revealed rapid aortic expansion, carotid tortuosity, and widespread loss of medial elastic fibers in the aortic wall. Based on these findings, we concluded that fluoroquinolone therapy had most likely caused the AA. Of course, this does not definitively diagnose fluoroquinolones as the cause of rapid aortic expansion, and as mentioned in the Limitations section, the possibility of an unspecified hereditary connective tissue disorder cannot be ruled out. However, our interpretation prioritized previously documented environmental triggers, particularly the administration of antibiotics that have been reported to induce similar clinical manifestations. Although the panel had a limited scope, we believe that the strong phenotypic similarity to previously reported antibiotic-associated cases, combined with the temporal relationship between drug exposure and symptom onset, strongly supports an environmentally acquired etiology in this case rather than an as-yet-undetected monogenic cause.

Three years ago, no cause was found for the patient's fever and elevated inflammatory markers, but fluoroquinolone therapy was effective in resolving her symptoms. Thus, the use of fluoroquinolones was considered justifiable despite the potential risk of aortic events. However, because these agents are reportedly associated with an increased risk of AAs/ADs, indiscriminate use of fluoroquinolones should be avoided in the management of infectious disease.^4^^,^^5^ Furthermore, given that even the resected specimen of the normal-diameter aortic arch showed severe loss of medial elastic fibers in this case, it is considered that the risk of aortic events in other areas will be higher than normal in the future. Although the aortic arch has already been prevented by aortic arch replacement, it will be necessary to carefully monitor the progression of events in the aorta distal to the descending thoracic aorta, given the tendency for the thoracoabdominal aorta to expand in the future.

To our knowledge, this is the first patient report to document the longitudinal changes in an AA following fluoroquinolone therapy, with a 30-mm aortic expansion over the span of 3 years. When fluoroquinolones are prescribed to patients with multiple risk factors, it is crucial to monitor them for rapid aortic expansion.

### Limitations

**Figure E3:**
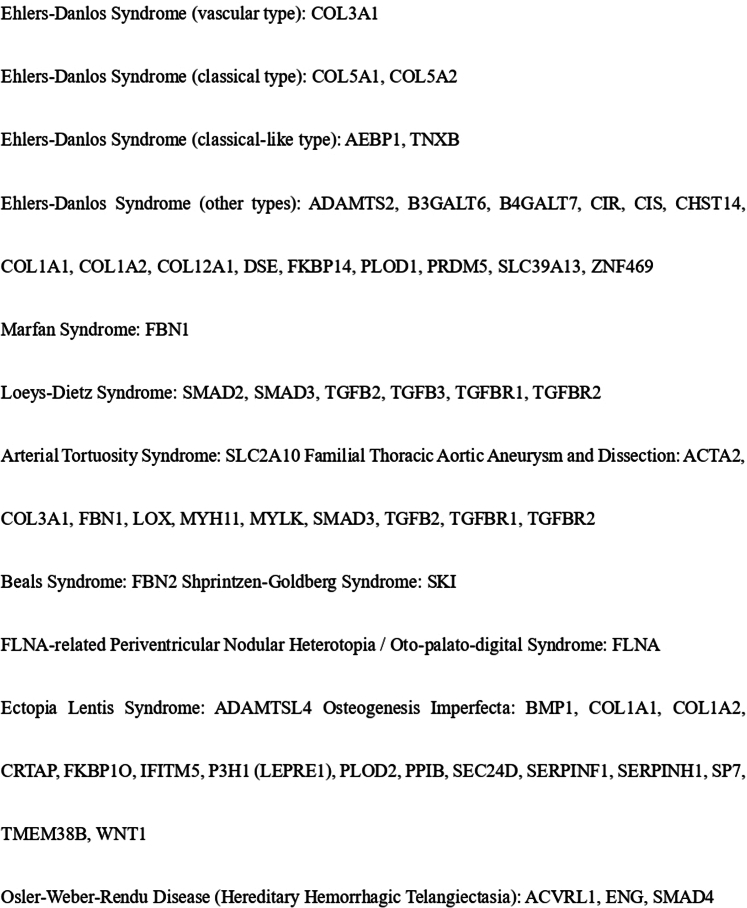
The genes included in our panel.

We analyzed more than 50 genes that have been reported to date to be associated with heritable connective tissue diseases, including those responsible for known syndromic and nonsyndromic aortopathies. Specifically, the genes included in our panel are known to be associated with a range of disorders (Figure E3). Variants located in genes not included in our panel, or within noncoding regions such as untranslated regions or deep intronic sequences, could theoretically be responsible for the observed phenotype.

## Conclusions

Because rapid aortic expansion has been observed after fluoroquinolone therapy, patients with predisposing risk factors who are prescribed these antibiotics must be carefully monitored.

## Conflict of Interest Statement

The authors reported no conflicts of interest.

The *Journal* policy requires editors and reviewers to disclose conflicts of interest and to decline handling or reviewing manuscripts for which they may have a conflict of interest. The editors and reviewers of this article have no conflicts of interest.
